# CT volume analysis in living donor liver transplantation: accuracy of three different approaches

**DOI:** 10.1186/s13244-023-01431-8

**Published:** 2023-05-15

**Authors:** Yerkezhan Kalshabay, Zhamilya Zholdybay, Michele Di Martino, Ulykbek Medeubekov, Dinara Baiguissova, Akmaral Ainakulova, Maksat Doskhanov, Bolatbek Baimakhanov

**Affiliations:** 1 Kazakh National Medical University Named After S.D. Asfendiyarov, Almaty, Republic of Kazakhstan; 2 National Scientific Center of Surgery Named After A.N. Syzganov, 51 Zheltoksan Street, A05F0D2 Almaty, Republic of Kazakhstan; 3 Department of Radiological, Oncological and Pathological Sciences, Sapienza University of Rome, Rome, Italy

**Keywords:** CT volumetry, Graft weight, Living donor liver transplantation (LDLT)

## Abstract

**Objectives:**

The aim of this retrospective study is to compare and evaluate accuracy of three different approaches of liver volume quantification in living donor transplantations.

**Methods:**

This is a single-center, retrospective study of 60 donors. The total and right lobe liver volumes were analyzed in the portal-venous phase by two independent radiologists who estimated the volumes using manual, semi-automated and automated segmentation methods. The measured right lobe liver volume was compared to the real weight of the graft after back-table examinations.

**Results:**

The mean estimated overall liver volume was 1164.4 ± 137.0 mL for manual, 1277.4 ± 190.4 mL for semi-automated and 1240.1 ± 108.5 mL for automated segmentation. The mean estimated right lobe volume was 762.0 ± 122.4 mL for manual, 792.9 ± 139.9 mL for semi-automated and 765.4 ± 132.7 mL for automated segmentation. The mean graft weight was 711.2 ± 142.9 g. The manual method better correlated with the graft weight (*r* = 0.730) in comparison with the semi-automated (*r* = 0.685) and the automated (*r* = 0.699) methods (*p* < 0.001). The mean error ratio in volume estimation by each application was 12.7 ± 16.6% for manual, 17.1 ± 17.3% for semi-automated, 14.7 ± 16.8% for automated methods. There was a statistically significant difference between the mean error ratio of the manual and the semi-automated segmentations (*p* = 0.017), and no statistically significant difference between the manual and the automated applications (*p* = 0.199).

**Conclusion:**

Volume analysis application better correlates with graft weight, but there is no obvious difference between correlation coefficients of all three methods. All three modalities had an error ratio, of which the semi-automated method showed the highest value.

**Critical relevance statement:**

Volume analysis application was more accurate, but there is no drastic difference between correlation coefficients of all three methods.

**Graphical abstract:**

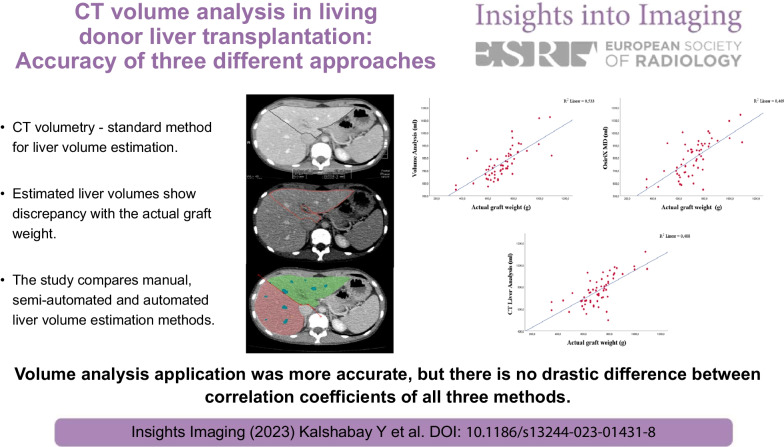

## Key points

- CT volumetry is the standard method for preoperative estimation of liver volume.
- There is a discrepancy in the estimated liver volume with graft weight.
- Manual, semi-automated and automated liver volume estimation methods were evaluated and compared.

## Introduction

Living donor liver transplantation (LDLT) is a standard procedure for patients with end-stage liver diseases. They are especially common for countries which are poor in cadaveric donors [1–5]. The precision in preoperative evaluation of a donor's liver volume plays crucial role for both the donor and a recipient. For the donor’s safety the remnant liver volume should be no less than 30–40% of total liver volume [6, 7]. At the same time, the graft-to-recipient weight ratio should be at least 0.8% in order to prevent post-transplant complications causing small-for-size syndrome in the recipient [8, 9].

Computer tomographic (CT) volumetry is the standard method for preoperative estimation of liver volume [10]. In 1970 Heymsfield et al. [11] was the first to estimate liver volume prior to operation. Since then, many software packages using manual, automated and semi-automated methods for measuring liver volume have been developed [12].

The manual method is the standard in volumetry, but it is time-consuming and heavily depends on the precision of the observer. With the development of semi-automated and automated technologies, some studies have shown that they provide acceptable liver volume estimations and dramatically reduce the amount of time required for evaluation [13, 14]. However, there is a tendency to discrepancy between liver volume estimations, conducted using any of the above methods, and the actual intraoperative graft weight [13, 15–19].

There are limited number of studies on the accuracy of these methods. Most studies compare results of only one or two methods with the graft weight [6, 7, 12–14, 20–28]. In our study, we include three different approaches for a more objective analysis of each method’s accuracy.

The aim of this retrospective study is to compare and evaluate accuracy of three methods: manual (Volume analysis), semi-automated (OsiriX MD) and automated segmentation (CT liver analysis) in measuring the right lobes of related donors’ livers, in relation to the actual graft weight in LDLT.

## Methods

This study was approved by the Ethics Committee of the Kazakh National Medical University named after S.D. Asfendiyarov (No. 3 (109) from 31.03.2021 year). Due to the retrospective nature of this study, the written informed consent was waived by the ethics committee and was not used in the study.

### Study population

**Fig. 1:**
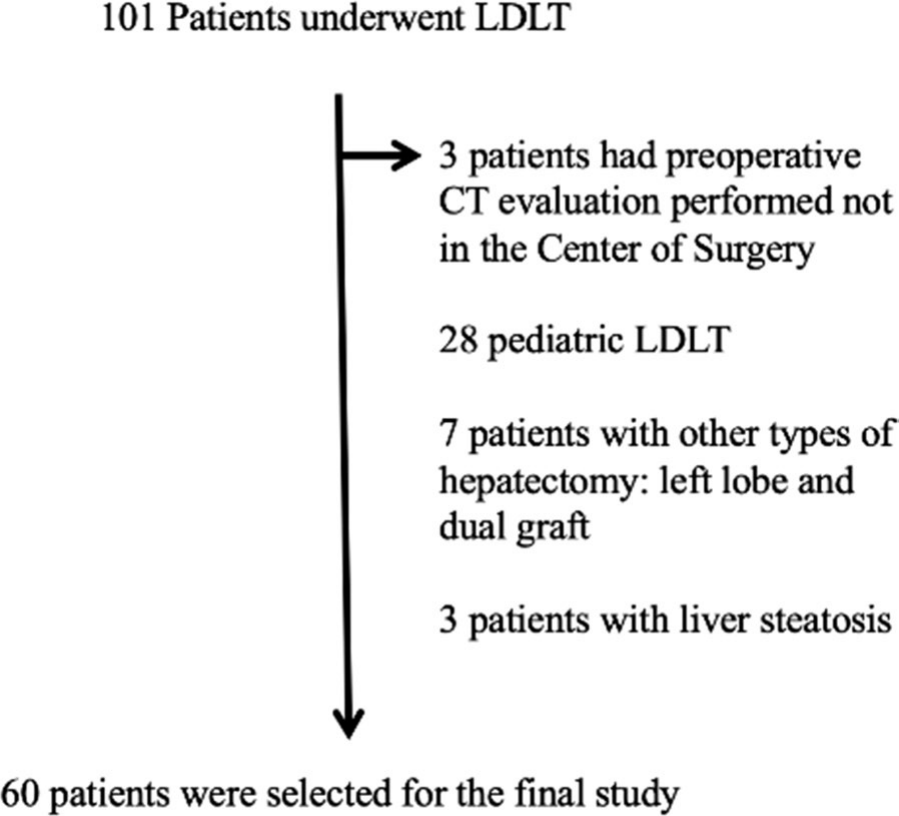
Flowchart of the study population

One hundred and one LDLT were performed in the Center of Surgery between 2018 and 2021, of which 71 were performed on adults. Among these, 60 LDLT had preoperative CT evaluation of liver volume conducted in the Department of Radiology and comprised the final study population (Fig. 1). There were 60 related liver donors (39 males and 21 females) with mean age of 28.8 ± 7.7 years. Inclusion criteria for this study were adults who underwent related living transplantation with right hepatectomy with no less than 35% of remnant liver volume. Patients who had preoperative CT evaluation conducted in other centers were excluded. All donors had a healthy liver. Patients with steatosis were excluded.

### CT imaging

Multiphasic CT was performed in the cranio-caudal direction using a 160-slice MDCT scanner (Canon Aquilion, Prime SP) with detector configuration: 0.5 × 80; tube voltage: 120 kVp; tube current: 63 mAs; gantry rotation time: 0.5 s; pitch: 0.637; scan time: 4–11 s and slice thickness in the axial and coronal planes: 5 mm (pre-contrast) or 3 mm (post-contrast) with no interslice gap. A soft tissue B20 kernel was used in all cases. All patients received 1.6 mL/kg of body weight (corresponding to 560 mg Iodine/kg) of a nonionic, iso-osmolar dimeric contrast medium (Iodixanol, Visipaque 320, GE Healthcare, Inc., Milwaukee, WI). Pre-warmed contrast medium (CM) was administered intravenously using a dual-chamber mechanical power injector at a rate of 4 mL/s through an 18-gauge IV catheter inserted into an antecubital vein. All injections were followed by a 30-mL saline flush administered at the same injection rate. After acquisition of an anteroposterior digital scout radiograph, patients were scanned craniocaudally from the dome of the liver to the iliac crest before and after intravenous contrast medium administration. Images were obtained during the hepatic arterial, portal-venous and delayed phases (25–40, 70 and 180 s, respectively, after the start of contrast medium injection).

The scan delay before initiation of hepatic arterial phase imaging was determined by means of bolus tracking with automated scan triggering. Arterial phase scanning began automatically 18 s after a trigger threshold of 150 Hounsfield units (HU) was reached in the supraceliac abdominal aorta.

Portal-venous dataset from all examinations was transferred from Picture Archiving and Communication System (PACS), and volume of the right lobe of the liver was calculated using three methods: Vitrea software which includes two different applications for manual segmentation (Volume analysis) and automated segmentation (CT liver analysis), and semi-automated software (OsiriX MD).

The estimation was carried out in two stages: (1) calculation of the total liver volume and (2) calculation of the left lobe plus segment I in order to establish the remnant liver volume. The volume of the right lobe of the liver equals to difference between the total volume and the left lobe of the liver, including segment I. Resection planes for liver segmentation passed through the right side of the middle hepatic vein and gallbladder bed. The resulting volume was further compared with the intraoperative weight of the graft. Estimated liver volumes are presented in milliliters (mL), graft weight in grams (g).

In order to assess and minimize inter-observer variation we included all estimations which were made by two independent observers. Two radiologists (Radiologist 1; Radiologist 2) with different levels of experience in abdominal radiology and CT volumetry (10 and 4 years, respectively) performed manual, semi-automated and automated estimations. The radiologists were uninformed of each other’s results.

Time needed for assessment of the total and residual liver volumes was measured from the moment of the first contour drawing until the last one using a stopwatch. Measured time included the correction of false-positive results for OsiriX MD and building a virtual resection line for CT liver analysis. Readers recorded the estimated time spent to measure each donor’s liver volume in minutes independently. The mean time of the two readers was taken for further analysis.

### Manual CT volumetry using Volume analysis application of the Vitrea software

**Fig. 2:**
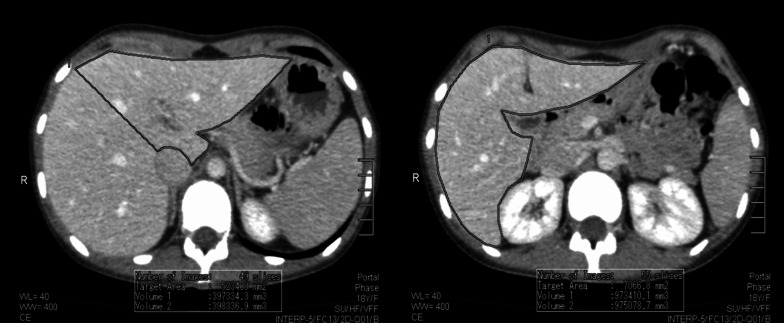
Manual CT volumetry of the liver by Vitrea software

On each axial scan, the contour of the liver was manually outlined with the mouse cursor using a pencil tool. The inferior vena cava, the portal vein with main branches and the gallbladder were excluded from the region of interest. Total liver volume and remnant liver volume were obtained by summing up the volumes on each section. The estimation of the volume included density of liver parenchyma; therefore, vessels were excluded. The results were saved as a screenshot (Fig. 2).

### Semi-automated CT volumetry using OsiriX MD software

**Fig. 3:**
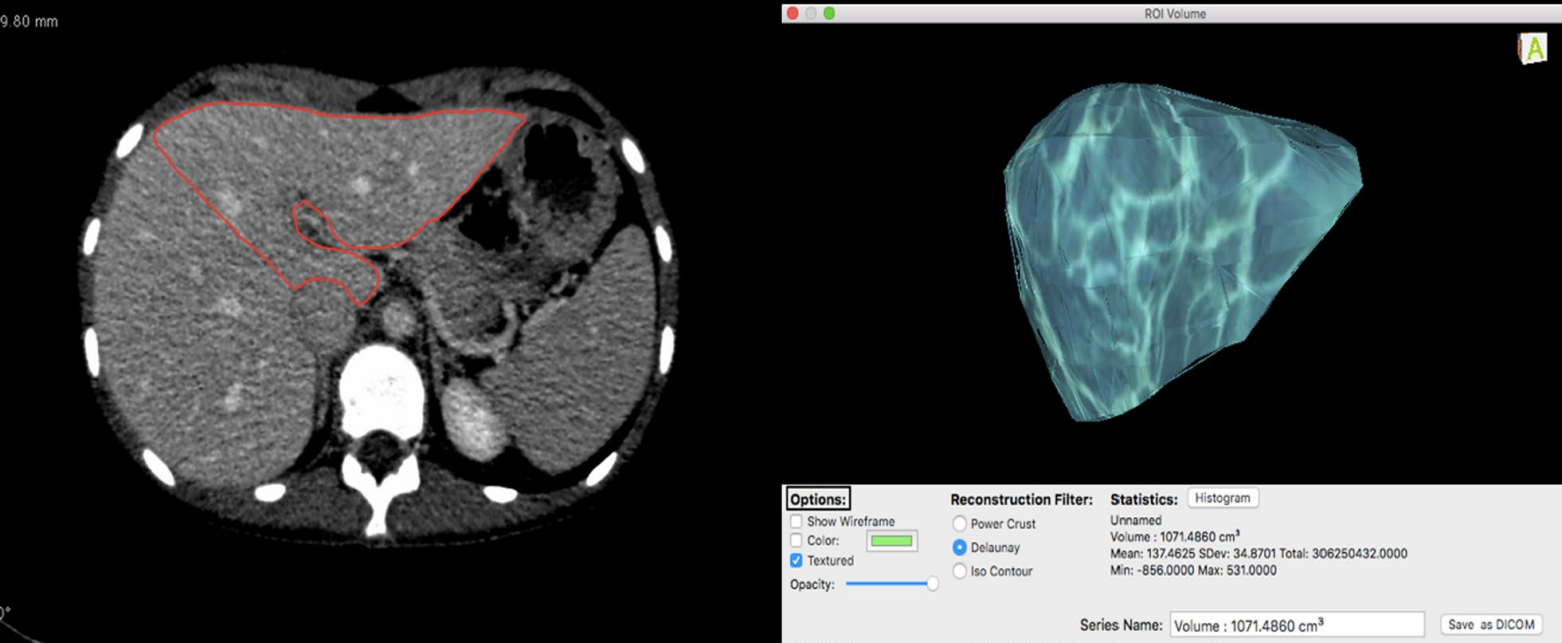
Semi-automated CT volumetry of the liver by OsiriX MD software

**Fig. 4:**
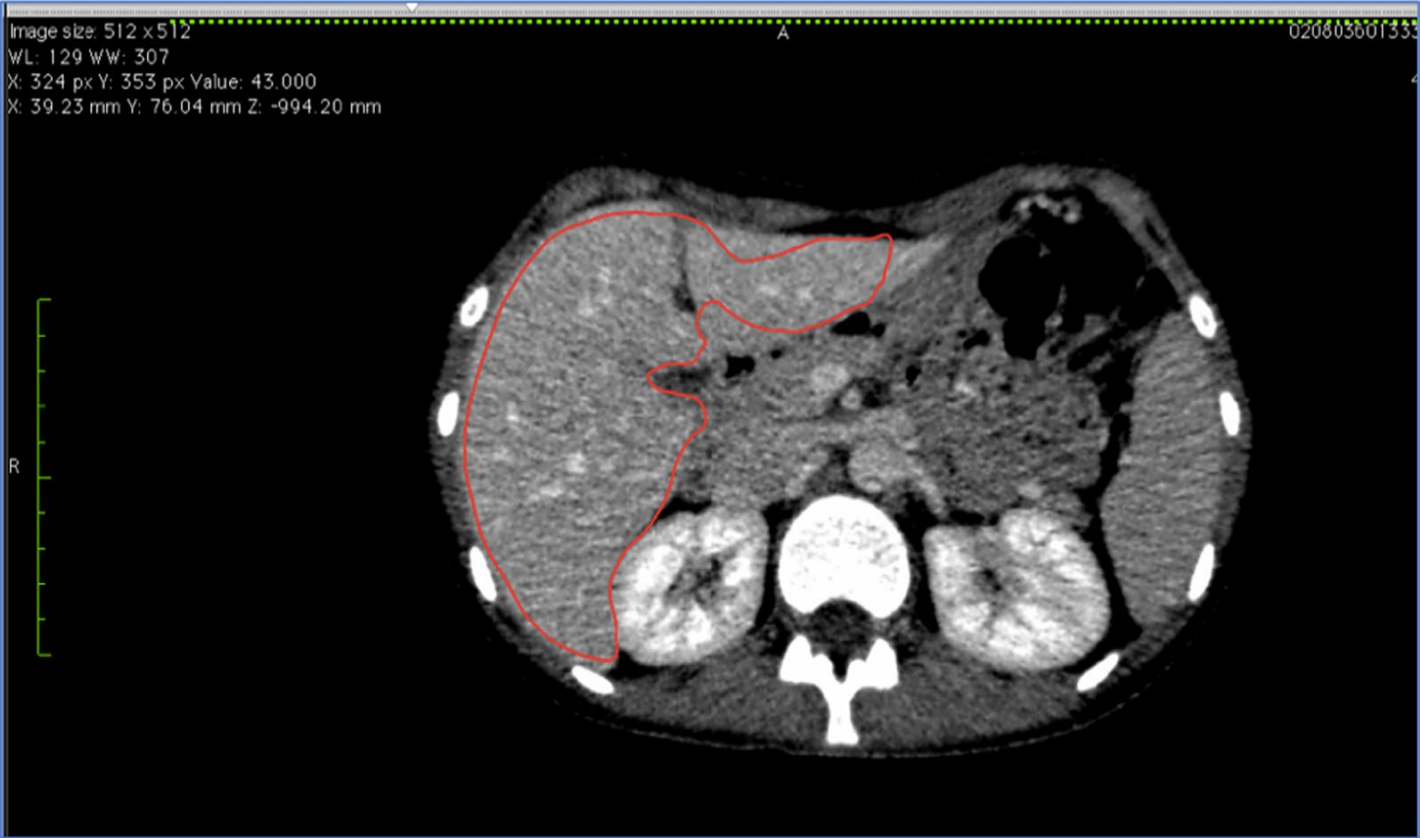
False-positive result acquired by OsiriX MD software*.* The contours automatically generated by the software (red line) do not correspond to the anatomical boundaries of the liver

On every third axial CT scan the region of the liver was traced, and the main vessels were excluded. Following, the program automatically constructed the missing parts. The results were saved as a screenshot (Fig. 3). Obtained false-positive isolated parts were corrected manually (Fig. 4).

### Automated volumetry using CT liver analysis application of the Vitrea software

**Fig. 5:**
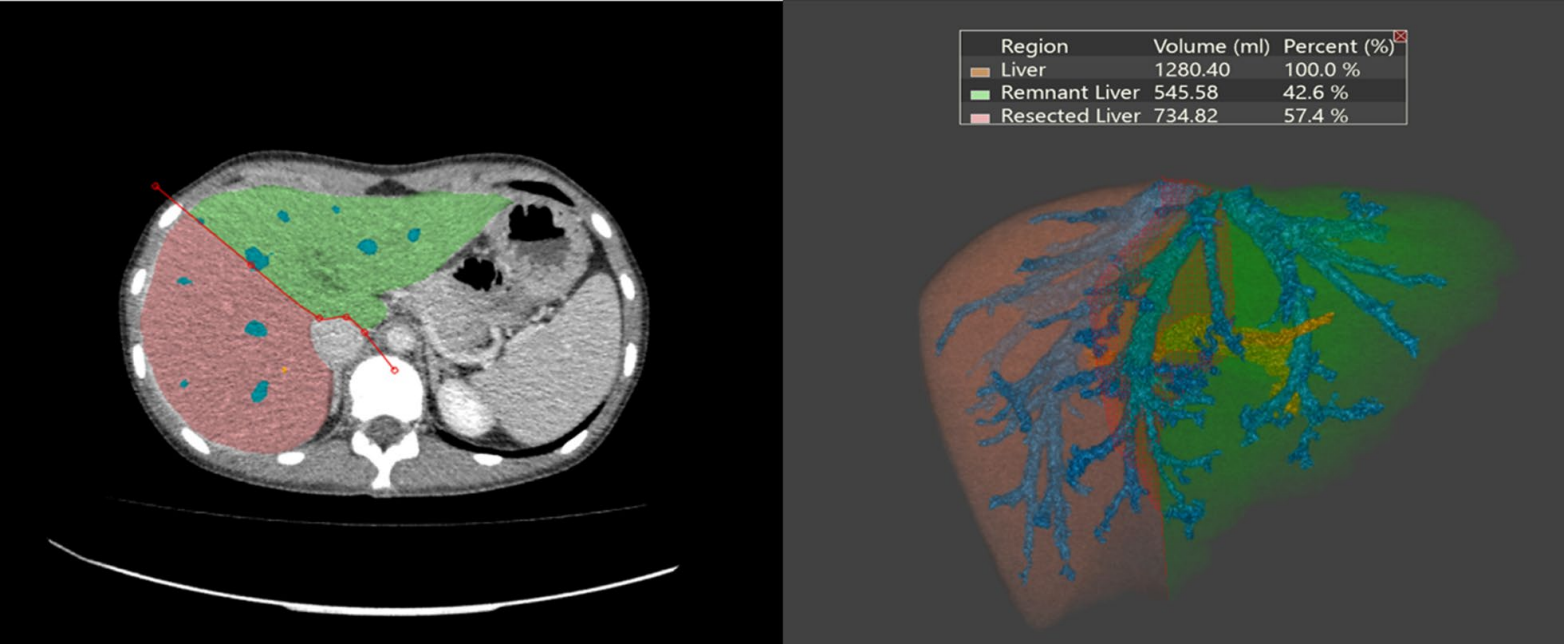
Automated CT volumetry and 3D resection plan by Vitrea software

**Fig. 6:**
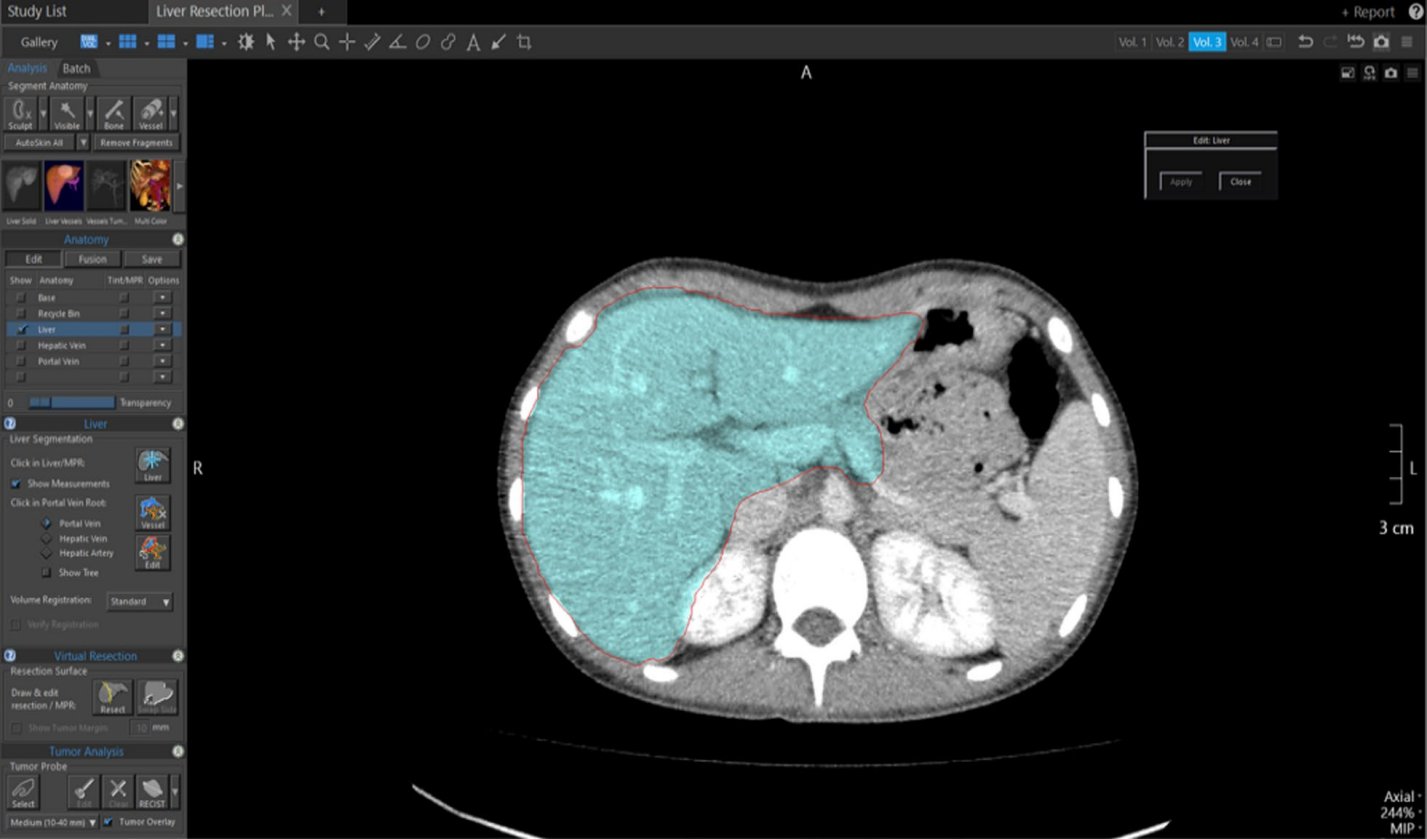
False-positive result acquired by CT liver analysis application. The liver parenchyma automatically generated by the software (blue color) includes parenchyma of the right kidney and pancreas

The application chose the border of the liver automatically. After finishing the segmentation on all slices, the application constructed a 3D model. After that, the liver was interactively segmented by a radiologist into a resected and remnant liver using a curved resection plane. Volumetric results of the overall liver, and resected and remnant parts, and intrahepatic vessels were calculated automatically and are demonstrated on the slide. The volume of intrahepatic vessels had been deducted (Fig. 5). Obtained false-positive isolated parts were corrected manually (Fig. 6).

### Intraoperative graft weight measurement

At the back table after resection, the graft was flushed by a surgeon with saline and histidine-tryptophan-ketoglutarate (Custodiol) solutions to remove blood. Afterward, the graft was weighed on electric scales.

### Statistics

For descriptive data analysis, graft weight and estimated liver volumes were presented as mean ± standard deviation (SD).

Pearson correlation was used to demonstrate correlation between graft weight and right lobe volumes estimated using Volume analysis application, OsiriX MD, CT liver analysis application. The correlation coefficient (*r*) was calculated.

Difference between estimated right lobe and graft weight, error ratio [15] in volume estimation by each program were calculated using special formulas:

$$\begin{aligned} & {\text{Difference}}\;\left( {{\text{mL}}} \right) = {\text{estimated}}\;{\text{right}}\;{\text{lobe}} - {\text{graft}}; \\ & {\text{Error}}\;{\text{ratio}}\;\left( \% \right) = \left( {{\text{estimated}}\;{\text{right}}\;{\text{lobe}} - {\text{graft}}} \right)/{\text{graft}}*{1}00\% ; \\ \end{aligned}$$

Boxplot was used to compare and show the difference and error ratio between graft and estimated volumes. The median, 25th and 75th percentiles were used to show results. A *p* value < 0.05 was considered to indicate statistical significance.

The paired t test was used to determine statistically significant differences in volumetric measurements of Radiologist 1 and Radiologist 2. The 95% limits of agreement were calculated.

The time spent on the volume estimation by each software program was presented as mean and range (minimum–maximum). Mann–Whitney *U* test was used to determine statistically significant differences in the lengths of used time of the three methods.

Univariate design by factorial ANOVA was used to find statistically significant relation between age, gender, body mass index (BMI), the quantity of the days between CT and surgery procedure, and the error ratio in volume estimations by both applications of Vitrea software and OsiriX MD software.

Statistical analyses were conducted using SPSS software (IBM corp., 28 version).

## Results

The mean age of the donors was 28.8 ± 7.8 years (min-18, max-51).

### Interobserver agreement

We did not find statistically significant differences between measurements performed with three approaches by Radiologist 1 and Radiologist 2 either of the total liver volumes: manual (*p* = 0.102), semi-automated (*p* = 0.462), automated (*p* = 0.506); or of the right lobe volumes: manual (*p* = 0.222), semi-automated (*p* = 0.101), automated (*p* = 0.061).

The mean difference of estimated total liver volumes between the radiologists was 4.6 ± 21.5 mL for manual, −6.5 ± 68.2 mL for semi-automated and −2.6 ± 30.1 mL for automated methods. The 95% limits of agreement were from –37.5 to 46.7 mL for manual, from –140.2 to 127.1 mL for semi-automated and from –61.6 to 56.4 mL for automated methods.

The mean difference of the estimation of the right lobe volumes was 3.4 ± 21.0 mL for manual, 4.5 ± 21.0 mL for semi-automated and 4.8 ± 19.7 mL for automated methods. The 95% limits of agreement were from -37.8 to 44.6 mL for manual, from –36.7 to 45.7 mL for semi-automated, from − 33.6 to 43.2 mL for automated methods.

### The measured liver volume

The mean estimated overall liver volume was 1164.4 ± 137.0 mL for Volume analysis; 1277.4 ± 190.4 mL for OsiriX MD; and 1240.1 ± 108.5 mL for CT liver analysis.

The mean estimated right lobe volume was 762.0 ± 122.4 mL for manual; 792.9 ± 139.9 mL for semi-automated; and 765.4 ± 132.7 mL for automated methods. The mean graft weight was 711.2 ± 142.9 g.

### Correlation between methods and graft weight

**Fig. 7:**
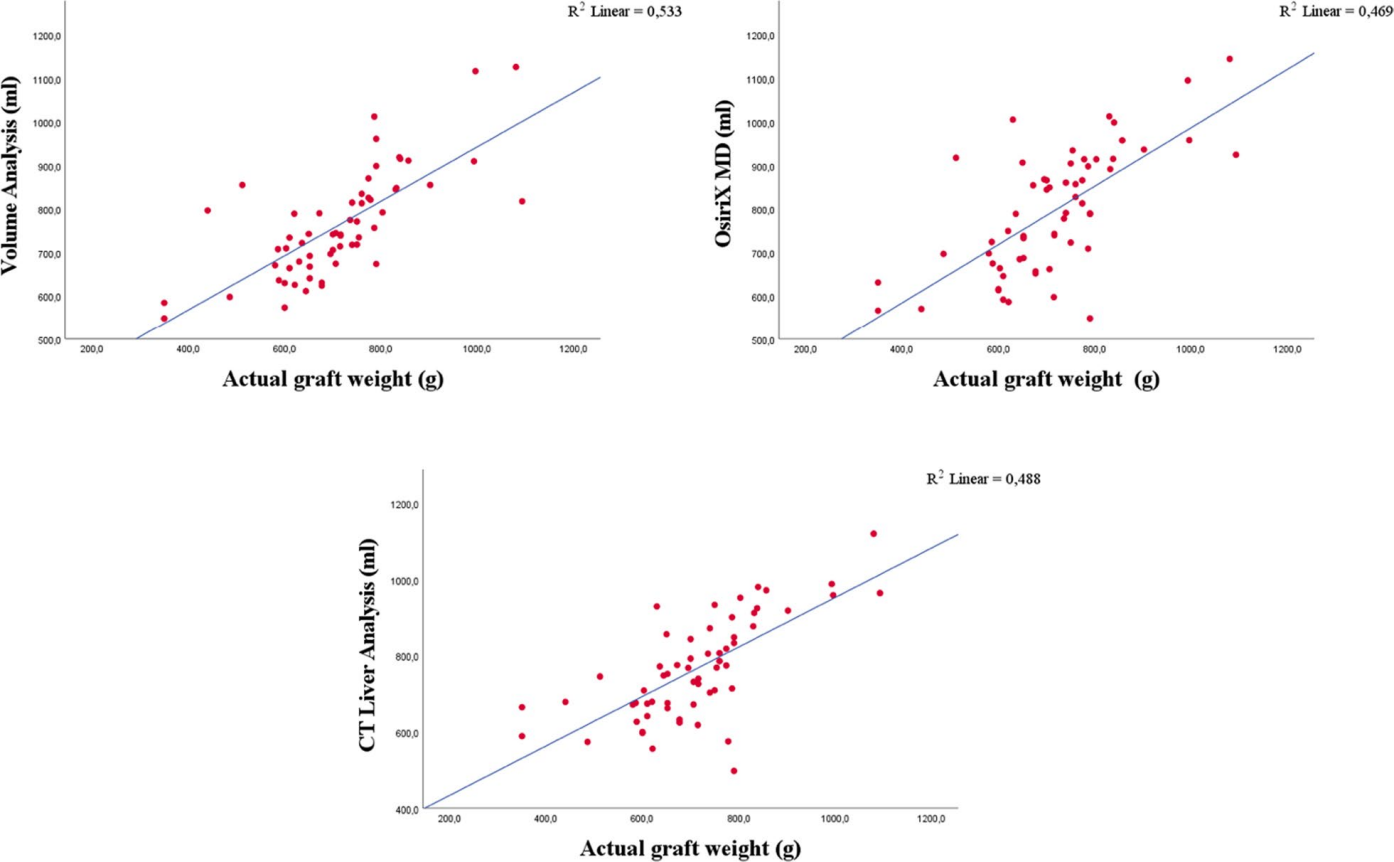
Correlation between right lobe volume, estimated by three methods and the graft weight. *r* = 0.730, *R*^2^ = 0.533 for Volume analysis; *r* = 0.685, *R*^2^ = 0.469 for OsiriX MD; *r* = 0.699, *R*^2^ = 0.488 for CT liver analysis

Pearson correlation and linear graph were used to estimate the association between graft weight and each estimated right lobe volume. We found that the volume of the liver’s right lobe estimated by Volume analysis better correlated with graft weight (*r* = 0.730; *p* < 0,001) in comparison with volume estimated by OsiriX MD (*r* = 0.685; *p* < 0,001) and CT liver analysis (*r* = 0.699; *p* < 0,001). *R*^2^ was 0.533 for volume analysis, 0.469 for OsiriX MD and 0.488 for CT liver analysis (*p* < 0,001) (Fig. 7). However, there was no significant difference between correlation coefficients of these three different measurement groups (*p* = 0.734; *p* = 0.631; *p* = 0.888).

### Comparative data

The mean error ratio was 12.7 ± 16.6% for Volume analysis, 17.1 ± 17.3%, for OsiriX MD, 14.7 ± 16.8% for CT liver analysis. We found a statistically significant difference between the mean error ratio of Volume analysis and OsiriX MD (*p* = 0.017), and no statistically significant difference between manual and automated applications of Vitrea software (*p* = 0.199). The mean difference was 50.8 ± 99.3 mL for Volume analysis, 81.7 ± 112.2 mL for OsiriX MD and 54.2 ± 107.5 mL for CT liver analysis (Table 1).

**Table 1:** Summary data of difference and error ratio according to three methods

Application	Difference (mL)	Error ratio (%)
*Volume analysis*		
Median	42.2	6.9
25th percentile	− 8.5	3.8
75th percentile	95.1	14.6
*OsiriX MD*		
Median	42.2	12.5
25th percentile	17.8	5.5
75th percentile	144.2	21.7
*CT liver analysis*		
Median	55.9	9.9
25th percentile	0.2	4.8
75th percentile	105.6	17.3

**Fig. 8:**
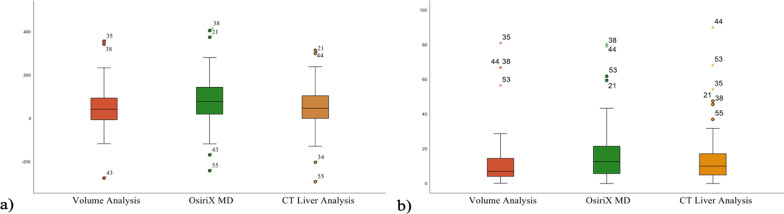
Difference of right lobe volume estimations (**a**) and error ratio (**b**) using the three methods. The middle lines are medians. The central boxes are value from 25 to 75th percentile

Boxplot shows the difference and error ratio by three methods (Fig. 8).

### The time consumption

The mean time spent on calculating total liver volume by Volume analysis was 26.7 min (range 20.0–33.8 min), calculating residual volume of the liver—13.8 min (range 10.1–18.7 min). To calculate total liver volume using the OsiriX MD software, including the correction of false-positive results, took at the mean 18.8 min (range 15.4–22.7 min), and it took 10.6 min (range 8.7–18.0 min) to calculate the residual liver volume with correction. CT liver analysis software calculated the total volume of the liver automatically in 1 min, but it took at the mean 9.3 min (range 4.1–15.2 min) to correct false-positive results and build a virtual resection line. The semi-automated and automated methods were significantly faster than the manual method in the mean estimation of total and residual volume of the liver (*p* < 0.05).

### Analysis of the relation between age, gender, BMI, waiting time and the error ratio

Univariate design by factorial ANOVA did not reveal any statistically significant relations between age (*p* = 0.682; *p* = 0.886; *p* = 0.898), gender (*p* = 0.325; *p* = 0.975; *p* = 0.467), BMI (*p* = 0.114; *p* = 0.441; *p* = 0.313), quantity of days between CT volumetry and surgery procedure (*p* = 0.341; *p* = 0.320; *p* = 0.459) and the error ratio of manual, semi-automated and automated methods.

## Discussion

Our study evaluated and compared the accuracy of three methods of liver volume estimation. It showed a comparable correlation between the calculations of liver volume made using all three approaches and the graft weight.

Despite the authors' reasoning that the semi-automated and automated methods are more accurate, we did not reveal a significant difference between correlation coefficients of the three different measurement groups. Even though the manual segmentation performed with volume application of Vitrea software is laborious and requires high precision from radiologists, it showed more positive correlation with the graft weight.

All three approaches had some error ratio. OsiriX MD demonstrated the highest error ratio. The difference in error ratio between two Vitrea applications was not statistically significant. Thus, to perform donor liver volumetry, it is preferable to use both applications of Vitrea software. In case if only the manual method is available, it is recommended to use it rather than the semi-automatic one, since the manual method is more accurate.

All in all, to estimate total and residual liver volumes by manual segmentation, radiologists spent approximately 11 min more than when using other approaches.

CT liver analysis application automatically calculated volume of the liver; however, sufficient time was needed to correct false-positive results, especially in cases where the density of the liver parenchyma matched that of adjacent organs, such as a spleen, pancreas and kidney. It is worth noting the advantages of this program in the form of virtual liver resection: a user-friendly interface that allows to significantly quicker perform a virtual resection and evaluate the residual volume of the liver.

Martel et al. [29] reported in their study that a discrepancy of about 5% between the calculated volume and the actual graft weight can influence the clinical outcome. For this comparison, binomial proportions and ward 95% confidence intervals were generated. Using measured volumetry as the comparative standard, estimated volumetry has been shown to lead to a clinically significant over- or underestimation in almost one-third of patients, potentially affecting clinical decision-making for preventing liver insufficiency or ‘small-for-size syndrome.’ These syndromes include the remnant liver failing to sustain adequate organ function, leading to hyperbilirubinemia, coagulopathy, ascites, encephalopathy and hypoalbuminemia and ultimately to postoperative death.

There are some factors which affect the difference and estimation error between the preoperative liver volume and the actual graft weight.

Some authors investigated inter-observer variability between readers with different levels of experience and included medical students in their final year of medical school and radiologists without or with limited experience with the software. However, all participants were trained in liver anatomy and use of software before [20, 28, 30]. These studies did not reveal statistically significant differences between the measurements and demonstrated that CT volumetry can be performed by readers with different levels of experience, but they should be adequately trained and be aware of the anatomical landmarks of liver resection. In our study, volumetry using three methods was performed by two radiologists with different levels of experience. No statistically significant differences were found between the calculations.

In our study, we performed volumetry on 3-mm slices on each software. It is believed that the thinner the cut, the more accurate the results, since the likelihood of false-positive results is reduced [31]. The disadvantage of such a study is the large amount of time required. Moreover, Mayer et al. [32] in his study, provide data that there are no statistically significant differences between the examined liver volume made with low slice thickness (< 3 mm) and high slice thickness (> 3 mm).

Radtke et al. [33] studied the effect of contrast agents on CT examination in his studies. Authors implied that the contrast agent has the potential to increase the hepatic intravascular water content. Although he found that the unenhanced phase provided more accurate volumetric results than the study in the portal-venous phase, there was still a 14% error ratio. The author also mentioned that the resection line passed through the middle hepatic vein performed in the portal-venous phase due to the gold standard. In the current study, we investigated the portal-venous phase and believe that the anatomical visualization and extraction of vessels in this phase are more important factors.

Some of the studies compared estimated liver volume and graft weight with and without blood [21]. In the present study, the weight of the actual graft was measured intraoperatively after draining out the blood. During estimations of liver volume in each program, vessels were excluded. The estimation of liver volume by Volume analysis included the threshold of liver density. CT liver analysis application calculates volume of intrahepatic vessels automatically. During estimations made by OsiriX MD only main vessels were excluded, which probably led to the highest error ratio.

Satou et al. [7] in their study discussed that the difference between the actual graft weight and the preoperative calculation is partly due to the loss of blood contained in the graft. Dehydration induced by high osmotic preservative solution potentially decreases graft weight. Hiroshige et al. [15] immersed rat liver in University of Wisconsin solution (UW solution) and ascribed the weight loss to dehydration induced by the high osmotic preservative solution; later Kayashima et al. [34] subsequently demonstrated the weight loss of a human liver graft while flushing with UW solution. Mussin et al. [22] discussed that contrary to the previous studies that used UW solution, when they used histidine-tryptophan-ketoglutarete solution as the organ perfusion solution during flushing it prevented tissue dehydration. Custodiol has a lower osmolarity (310 mOsm/L vs 320 mOsm/L), lower sodium and lower potassium content than the UW solution. In our study, the graft was flushed by Custodiol solutions and we also think that there is less influence on the graft weight.

In our study, we compared the liver volume and the actual graft weight according to the generally accepted estimation of 1.0 g/mL. In previous studies, the authors discuss that the liver density would be different than 1.0 g/mL [17, 28, 35]. This difference may increase if the patient has developed hepatic steatosis which would influence the discrepancy [28, 36, 37]. To get more accurate calculations, we excluded patients with liver steatosis.

Additionally, the difference between the preoperative estimation of the liver volume and the actual graft weight may be explained by the difference between the virtual line of liver resection and the real resection of the surgeon [32]. In all our cases, the virtual resection followed the right side of the middle hepatic vein and gallbladder bed. During surgery, millimetric deviations seen on the tracing of the midhepatic vein may cause discrepancies in the volumetric assessment [38]. Dello et al. [24] in their study mentioned that hepatopancreatobiliary surgeons should routinely perform CT volumetry during the preoperative assessment of patients undergoing a major liver resection*.* Yonemura et al. [39] discussed that the “mismatch” of the virtual transection and intraoperative surgical planes at the time of the donor hepatectomy constitutes one of the most troublesome sources of error. We did not reveal comparative study involving a radiologist and a surgeon operating a donor. And we did not include surgeons’ estimations in this study, as this was not the aim of our work. However, we agree with all statements; despite the same anatomical landmarks, virtual resection may differ slightly from the real one and thus affect the discrepancy. We are convinced that virtual resection is preferable to be performed by surgeons operating the donor in planning transplantation to obtain more accurate measurements.

There have been studies where patients' age was affecting overestimation [34, 39]. Our findings suggest that there is no statistically significant relation between age and error ratio of estimated volume. Factors such as gender, BMI, the quantity of days between the CT volumetry and the surgery procedure also did not show statistically significant relation.

Our study had several limitations. Firstly, the study only included right hepatectomy. Donors with other resections were not studied. Secondly, the study was conducted only by radiologists, and surgeons were not involved in the CT volumetry of donors’ livers.

In conclusion, Volume analysis application better correlates with graft weight, but there is no obvious difference between correlation coefficients of all three methods. All three approaches showed some error ratio; however, both Volume analysis and CT liver analysis applications of Vitrea were more accurate. Thus, manual and automated segmentation methods are preferable in LDLT due to higher level of accuracy.

Further studies are needed to investigate intra- and intersoftware variability, to optimize CT volumetry in order to reduce discrepancies between the estimated liver volume and the actual graft weight.

## Data Availability

The datasets used and/or analyzed during the current study are available from the corresponding author upon reasonable request.

## Abbreviations

3D: Three-dimensional
BMI: Body mass index
CM: Contrast medium
CT: Computer tomography
HU: Hounsfield units
LDLT: Living donor liver transplantation
MDCT: Multidetector computer tomography
PACS: Picture Archiving and Communication System
UW solution: University of Wisconsin solution
